# Unraveling crop enzymatic browning through integrated omics

**DOI:** 10.3389/fpls.2024.1342639

**Published:** 2024-02-02

**Authors:** Chunkai Wang, Lin Meng, Guochao Zhang, Xiujun Yang, Bingwen Pang, Junjie Cheng, Bing He, Fushan Sun

**Affiliations:** ^1^ Key Laboratory of Tobacco Biology and Processing, Ministry of Agriculture, Tobacco Research Institute, Chinese Academy of Agricultural Sciences (CAAS), Qingdao, China; ^2^ Institute of Germplasm Resources and Biotechnology, Jiangsu Academy of Agricultural Sciences, Nanjing, China

**Keywords:** enzymatic browning, ROS, PPO activity, multi-omics, postharvest, oxidative stress

## Abstract

Enzymatic browning reactions, triggered by oxidative stress, significantly compromise the quality of harvested crops during postharvest handling. This has profound implications for the agricultural industry. Recent advances have employed a systematic, multi-omics approach to developing anti-browning treatments, thereby enhancing our understanding of the resistance mechanisms in harvested crops. This review illuminates the current multi-omics strategies, including transcriptomic, proteomic, and metabolomic methods, to elucidate the molecular mechanisms underlying browning. These strategies are pivotal for identifying potential metabolic markers or pathways that could mitigate browning in postharvest systems.

## 1 Introduction

Postharvest crops remain biologically active to sustain physiological functions and metabolic processes. However, this continued vitality renders them vulnerable to abiotic stresses during storage and processing phases, which often leads to enzymatic browning. Such browning not only diminishes the nutritional value, sensory appeal, and safety of the produce but also poses a significant challenge within the postharvest processing sector (Tinello and Lante, 2018). The escalating prevalence and severity of browning during storage and processing underscore the critical need to identify the mechanisms that bolster crop resilience to browning hazards.

During postharvest handling, crops undergo significant biochemical and metabolic transformations that lead to the accumulation of reactive oxygen species (ROS) and subsequent membrane disruption. The biochemical underpinnings of enzymatic browning involve the oxidation of phenolic substrates by oxidases to form ortho-quinone compounds. These compounds then polymerize with various substrates, culminating in the formation of brown pigments (Paudel et al., 2020). Prior studies have established that the incidence of enzymatic browning in harvested crops is associated with multiple stress factors, including senescence, desiccation, chilling injury, pathogen infection, mechanical damage, heat stress, and other processes (Yi et al., 2009). Commercially, a range of postharvest strategies is implemented to mitigate these effects, including maintaining low temperatures, modifying atmospheric conditions, and applying chemical treatments. These strategies are all aimed at reducing respiration rates, delaying senescence and browning, inhibiting pathogen proliferation, and preserving crop quality.

Identifying new compounds relevant to browning and elucidating their biosynthetic pathways in postharvest crops present significant challenges. Over the last two decades, omics research has made substantial strides across various biological and chemical fields, mainly owing to rapid advancements in high-throughput nucleic acid sequencing (e.g., next-generation sequencing, NGS) and the identification of proteins and metabolites through diverse mass spectrometry techniques (He et al., 2022). More recently, a paradigm shift has occurred in omics research based on the increasing application of these methods for evaluations of harvested crops processing and storage, with studies transitioning from single-omics to integrated multi-omics approaches (Yun et al., 2019; Sirangelo et al., 2022). This transition has inspired many integrated multi-omics studies, resulting in the exponential growth of omics-based research on the browning reaction (Liu et al., 2022b). Therefore, this review aims to consolidate current knowledge on integrated omics strategies, including transcriptomic, proteomic, and metabolomic analyses, and explore how these omics techniques contribute to a deeper understanding of the molecular mechanisms underlying the browning of harvested crops during the postharvest period.

## 2 Enzymatic browning during postharvest crop processing

**Figure 1:**
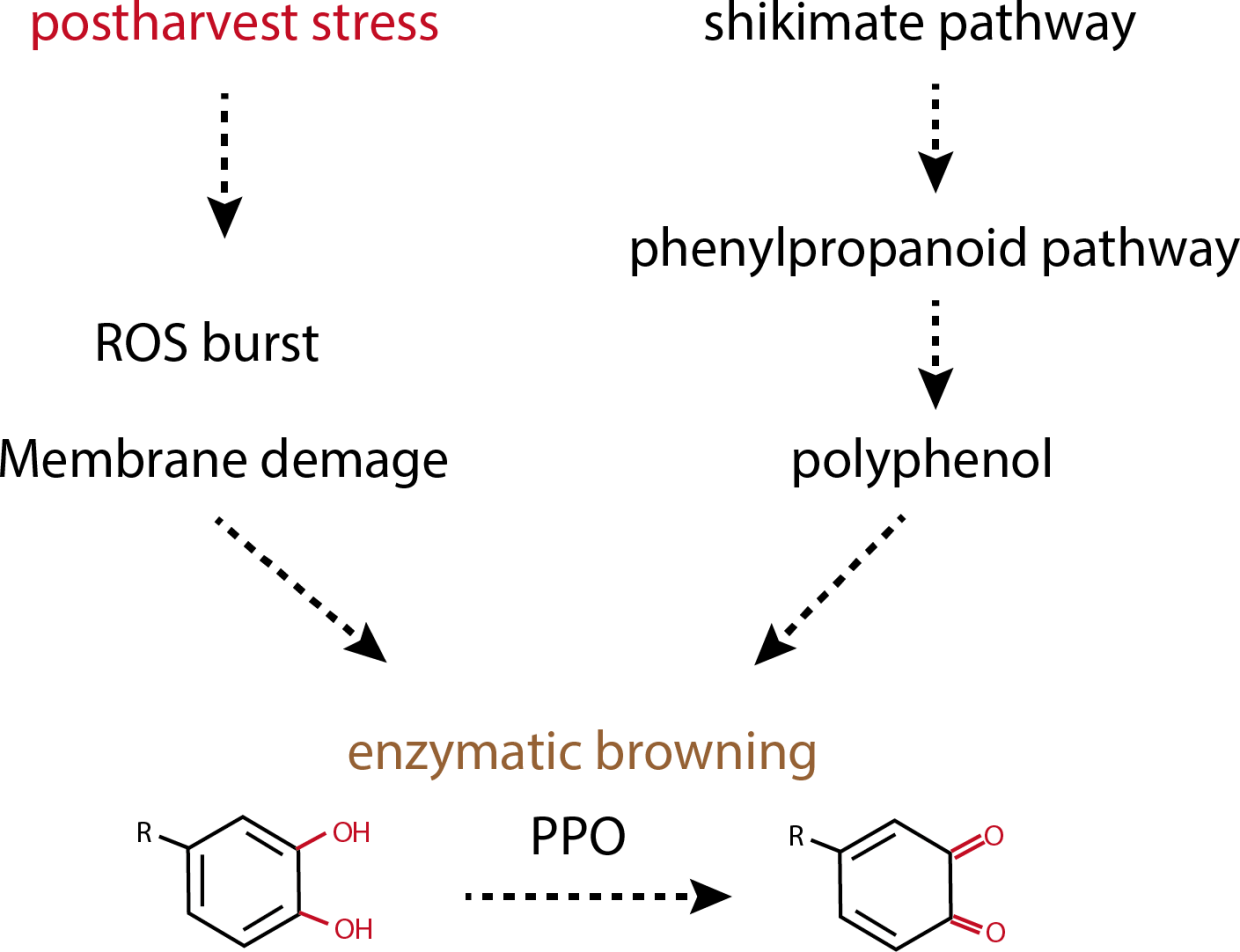
Biochemical mechanism of enzymatic browning during postharvest.

Several studies have established that is primarily accountable for the discoloration observed in crops during storage and processing (**Figure 1**). This browning occurs when crop polyphenols, or phenolic molecules, are oxidized by specific enzymes in the presence of oxygen, leading to the formation of quinones, which subsequently undergo chemical polymerization, resulting in a brown coloration (Parveen et al., 2010; Sui et al., 2023). Crops present a diverse array of phenolic compounds and oxidizing enzymes and thus are predisposed to rapid browning upon slicing, crushing, or any form of processing (Hamdan et al., 2022). The enzymatic browning mechanism is a process in which enzymes in the cytoplasm act upon substrates, mainly polyphenols located in plastids. This reaction does not occur in fresh fruit and vegetable due to separation of their enzymes from its substrate by cell compartments. During the handling of crops, tissue damage may cause plastids to rupture, facilitating the interaction between oxidase enzymes and their polyphenolic substrates, and thereby triggering the browning reaction (Sui et al., 2023).

### 2.1 Substrates of enzymatic browning

Phenolic compounds, widely recognized as polyphenols, are prevalent chemical constituents in a variety of fruits and vegetables and play a pivotal role in enzymatic browning by acting as substrates for the enzymes that catalyze this reaction (Singh et al., 2016). These compounds belong to a class of secondary metabolites synthesized via complex and irreversible pathways, notably the shikimate and phenylpropanoid pathways, which are exclusive to plants. Such pathways contribute to plant defense mechanisms against herbivores, microbial pathogens, invertebrate pests, and environmental stressors (Sun et al., 2022).

The structure of phenolic compounds is characterized by an aromatic ring bearing one or more hydroxyl groups, and their biosynthesis is primarily routed through the shikimate pathway, which leverages intermediates from carbohydrate metabolism (Singh et al., 2017). The oxidation potential of monocyclic phenolics is greatest for compounds with 2,4,5-trihydroxy substituents and lowest in monophenols. Typically, a wide range of 1,2-dihydroxyarenes, also referred to as ortho-dihydroxyphenols, are particularly prone to oxidation by polyphenol oxidase (PPO) due to the ortho positioning of the hydroxyl groups, which facilitates oxidation (Parveen et al., 2010).

The oxidation process mediated by PPOs is significantly influenced by the characteristics of the phenolic side chain, including the nature and number of hydroxyl groups and their placement on the aromatic ring (Winters et al., 2008). These structural attributes also determine the extent to which these compounds can undergo indirect oxidation through interactions with PPO reaction products. The substrate specificity of PPOs varies depending on the plant species and isoform, with a generally higher affinity for diphenols over monophenols. Consequently, polyphenols are recognized as the principal contributors to enzymatic browning in postharvest storage and processing of crops.

### 2.2 Enzymes of enzymatic browning

Enzymatic browning is a biochemical process in which specific enzymes oxidize phenolic compounds to form quinones, which then non-enzymatically polymerize to produce brown pigments (Zhu et al., 2023). The chief enzyme implicated in this browning reaction is polyphenol oxidase (PPO), which is categorized into two main types: EC 1.10.3.1 (including o-diphenol oxygen oxidoreductase, diphenol oxidase, and catechol oxidase) and EC 1.14.18.1 (encompassing tyrosinase, cresolase, and monophenol monooxygenase) (Moon et al., 2020). PPO enzymes are located within the cytoplasm, while their phenolic substrates are typically housed within plastids. Tissue damage in plants triggers the migration of PPO enzymes from the cytoplasm to the plastids, facilitating their interaction with the substrates.

Ortho-quinones, the products of PPO activity, display electrophilic characteristics, making them prone to nucleophilic attacks from a variety of biomolecules, such as proteins, peptides, amino acids, water, and other polyphenols. These interactions lead to the formation of Michael-type adducts, contributing to the complexity of the browning pigments (Loizzo et al., 2012; Schieber, 2018). Additionally, peroxidase (POD, EC 1.11.1.7) is another thermostable enzyme that significantly contributes to oxidative browning reactions (Shrestha et al., 2020). Utilizing hydrogen peroxide (H2O2) as a cofactor, POD catalyzes the single-electron oxidation of a wide array of substrates, further promoting enzymatic browning. It simultaneously reduces hydrogen peroxide to water, thus participating actively in the browning process (Nokthai et al., 2010).

Therefore, both PPO and POD are instrumental in the enzymatic browning process. They not only share common substrates but also their concerted action on diphenolic substrates can lead to melanin formation, which is a key component of the browning phenotype in plants.

### 2.3 The influence of reactive oxygen species on enzymatic browning

Reactive oxygen species (ROS) are byproducts of cellular processes such as photosynthesis, respiration, and other metabolic activities. These include singlet oxygen (1O_2_), hydrogen peroxide (H_2_O_2_), superoxide anions (O_2_−), hydroxyl radicals (OH·), lipid peroxide (ROOR′), and alkyl radicals (R) (Li et al., 2019a). At low concentrations, ROS act as signaling molecules within plant cells, playing a pivotal role in various physiological functions. It can induce both reversible and irreversible oxidative post-translational modifications to proteins involved in many signaling cascades (Waszczak et al., 2015). However, when produced in excess, ROS can cause oxidative stress, which is detrimental to the organism.

High levels of ROS can induce senescence, compromising the integrity and functionality of cellular membranes. This interaction between ROS and cellular biomolecules can lead to their modification or inactivation, resulting in organelle dysfunction and structural cellular changes (Rezayian et al., 2019). During postharvest storage and processing, the accumulation of ROS and free radicals can trigger browning reactions. Specifically, peel browning is often attributed to the release of cellular contents following the loss of membrane permeability caused by ROS surges (Lin et al., 2020).

Controlling the ROS pathway is essential for reducing browning and maintaining the quality of fruits and vegetables after harvest. Plants can mitigate browning by enhancing the activity of antioxidative enzymes and increasing the antioxidant content within the fruit, thus counteracting the accumulation of ROS (Ali et al., 2019; Li et al., 2019a; Liu et al., 2021b). The ROS metabolic pathway is regulated by three primary antioxidant enzymes: superoxide dismutase (SOD), ascorbate peroxidase (APX), and catalase (CAT). These enzymes play a critical role in maintaining ROS balance and protecting plants against oxidative stress (Li et al., 2019b). SOD catalyzes the dismutation of O_2_^−^ into H_2_O_2_ and O_2_, while APX and CAT efficiently detoxify H_2_O_2_. By reducing ROS levels, these enzymatic ROS scavengers not only prevent oxidative damage but also inhibit enzymatic browning processes (Li et al., 2019c).

### 2.4 The role of intracellular membrane integrity in enzymatic browning

The integrity of the plasma membrane plays a pivotal role in preventing browning in postharvest produce. Compromised cell membrane integrity facilitates the contact between phenolic compounds and oxidative enzymes, catalyzing the enzymatic browning process (Ma et al., 2022). This degradation is often indicated by a shift from unsaturated to saturated fatty acids in the membrane, coupled with an increase in lipid peroxidation products and enhanced membrane permeability (Lin et al., 2016).

Phospholipids are essential components of cellular membranes, and enzymes such as Phospholipase D (PLD) and lipoxygenase (LOX) are critical in the breakdown of membrane phospholipids, an early sign of senescence and browning in harvested tissues (Li et al., 2009; Sun et al., 2020). PLD catalyzes the hydrolysis of phosphatidylcholine (PC) or phosphatidylethanolamine (PE) to produce phosphatidic acid (PA), whereas LOX initiates the peroxidation of membrane lipids. This peroxidation can lead to the deterioration of the cell membrane’s structural integrity, resulting in the disruption of the phospholipid bilayer and loss of cellular compartmentalization, which are critical events preceding browning (Trabelsi et al., 2012).

## 3 Omics technologies and postharvest crop browning studies

The browning reaction significantly impacts marketability during the storage and processing of postharvest crops. Unraveling the molecular basis of the browning reaction is key to understanding and developing effective strategies to inhibit it. Omics technologies, comprising transcriptomics, proteomics, and metabolomics, provide comprehensive insights when applied either individually or synergistically. These methodologies have been instrumental in assessing the effects of various postharvest treatments aimed at mitigating browning during storage and processing (**Table 1**).

**Table 1:** Recent articles on integrated omics analysis of crops, type of omics integration and major output.

Crop	Integrated omics	Major Output	Reference
Longan	Transcriptome Metabolome	Polymerization reaction of PAs and lignin monomers mediated by LACs/PRXs is induced by water-loss leading to the browning of longan pericarp.	(Liu et al., 2024)
Lilium bulb	Transcriptome	Phenol and fatty biosynthesis are responsible for browning and a complex hormone signaling network and most genes responsive to injury transcription factors significantly change.	(Wang et al., 2023a)
Fresh-cut apples	Transcriptome Metabolome	Selenium inhibits browning of fresh-cut apples by reducing membrane lipid degradation and increase gene expression of the antioxidant.	(Wang et al., 2023b)
Apple	Transcriptome Proteome Methylome	Methylated-*NCA1* and O-methyltransferase 1 (OMT1) significantly increased in apple browning	(Wang et al., 2022)
Flesh-cut eggplant	Transcriptome Metabolome	Chlorogenic acid act as the main browning substrate in fresh-cut eggplant	(Liu et al., 2022a)
Fresh-cut lettuce	Transcriptome Metabolome	6-Benzylaminopurine reduce browning by inhibiting phenolic-related metabolite biosynthesis, especially scopoletin.	(Liu et al., 2022b)
Morel	Metabolome	Tyrosine metabolism is involved in browning of morels during storage	(Gao et al., 2022)
Fresh-Cut Sand Pear	Transcriptome	Fresh-cut sand pear fruit enzymatic browning is due to the expression of *PbrPPO4* that was probably regulated by lncRNA *PB.156.1.*	(Fan et al., 2021)
Grape	Proteome	Browning is primary involved in phenylpropanoid biosynthesis, tyrosine metabolism, phenylalanine metabolism, oxidative phosphorylation metabolism, glutathione metabolism, peroxisome pathway, and fatty acid degradation	(Liu et al., 2021a)
Fresh-cut apples	Transcriptome	γ-aminobutyric acid reduce browning by regulating the genes expression related to the synthesis of browning enzymes and phenolic substances	(Zhao et al., 2021)
Grape	Proteome	The browning-related proteins are primarily involved in the phenylpropanoid biosynthesis, oxidative phosphorylation metabolism, peroxisome pathway and fatty acid degradation.	(Liu et al., 2021a)
Litchi	Transcriptome Proteome	Anthocyanin metabolism is involved in litchi pericarp browning	(Qu et al., 2021)
Flesh-cut eggplant	Transcriptome	Browning is involved in expression regulatory networks was set up based on tyrosine metabolism and phenylpropanoid biosynthesis	(Liu et al., 2021c)
Litchi	Long-read sequencing transcriptome	During the ‘browning’ stage, the expression of isoforms related to cell wall degradation, oxidation, and disease response was significantly up-regulated.	(Zhou et al., 2020b)
Pear	Transcriptome	DEGs indicates redox reaction, membrane lipid metabolism account for the browning disorder.	(Zhou et al., 2020a)
‘Nanguo’ pear	Proteome	Peel browning is primarily involved in the phenylpropanoid pathway, linoleic acid pathways, fatty acid biosynthesis pathway, glutathione metabolism pathway, photosynthesis pathway, oxidative phosphorylation pathway, and glycolysis pathway.	(Wang et al., 2017)
Fresh-Cut lettuce	Metabolome	Browning process kinetics is associated with a higher level of constitutive lysophospholipids and constitutive levels of caffeoylquinic derivatives,	(García et al., 2017)

### 3.1 The application of transcriptome in postharvest browning

Transcriptomics is the study of RNA expression profiles, including both coding and regulatory non-coding RNA sequences, within a given temporal context (Pandit et al., 2018; Luo et al., 2022). This analysis can be conducted through hybridization-based methods, such as microarrays, or sequence-based approaches, like direct cDNA sequencing. The browning process in postharvest produce involves significant changes in gene expression.

Early genetic studies focused on identifying key factors in the browning of postharvest fruits and vegetables, providing foundational insights into the underlying regulatory mechanisms (Coetzer et al., 2001). However, as whole-genome transcriptomic studies advanced, genes related to the browning process, including those involved in downstream signaling and anti-browning responses, have been somewhat neglected. Our current study employs transcriptome analysis of fresh-cut potato tubers to pinpoint critical genes implicated in browning, highlighting the roles of plant hormone biosynthesis, signaling molecules, and respiratory burst oxidase in this process (Wang et al., 2023a).

Senescence, a crucial stage in plant development, is intimately connected with browning. Zhou et al. (2020b) utilized long-read sequencing technology to identify a two-phase browning pattern in postharvest litchi, distinguishing the onset of senescence from the browning stage. Their findings underscored significant stage-specific biological pathway activations, such as cell wall degradation, oxidative processes, and disease responses during browning. Comparative transcriptomic analyses between browning-resistant and susceptible cultivars have revealed candidate genes and mechanisms involved in postharvest browning. A recent time-course study on fresh-cut eggplant employed transcriptome analysis to unravel the transcriptional regulation of browning, proposing two key regulatory networks related to tyrosine metabolism and phenylpropanoid biosynthesis (Liu et al., 2021c).

Transcriptomics has also been applied to understand how postharvest crops respond to anti-browning treatments. Zhao et al. (2021) explored how γ-aminobutyric acid (GABA) prevents browning in fresh-cut apples, finding that GABA modified the expression of genes related to the synthesis of browning enzymes and phenolic compounds. Similarly, the application of melatonin, an effective anti-browning agent, was shown to enhance the antioxidant system by regulating ROS-metabolism-related genes in a comparative transcriptome study (Min et al., 2023). Plant hormones, which play a critical role in regulating postharvest browning, have been studied as well; for instance, Liu et al. (2022b) demonstrated that 6-Benzylaminopurine (6-BA) effectively delayed browning in fresh-cut lettuce, with transcriptome analysis indicating a significant impact on phenolic-related metabolic pathways.

### 3.2 Proteome and its impact on postharvest browning studies

Proteomics investigates the abundance, expression, and interactions of proteins within organisms or cells. Unlike transcriptomic or genomic data, proteomic information provides a more direct understanding of the actual functional molecules in biological processes, as protein levels and activities cannot be fully predicted from RNA or DNA data alone (Mathabe et al., 2020). Proteins are central to the regulation and execution of nearly all biological functions, making proteomics essential for uncovering the molecular mechanisms that govern the development and browning of postharvest crops.

Recent proteomic studies have focused on gene expression and the accumulation of proteins during the storage and processing of crops (Feng et al., 2016). These studies have the potential to reduce postharvest losses due to browning by identifying and selecting crop varieties with enhanced tolerance to browning and other desirable quality traits. For instance, Qu et al. (2022) analyzed the proteome of postharvest mushroom fruiting bodies during storage, identifying 168 significantly regulated proteins involved in processes such as translation, carbohydrate metabolism, signal transduction, and amino acid metabolism. Their research also highlighted the role of AMPK and FOXO signaling pathways in the browning of mushrooms during storage. Ban et al. (2018) applied proteomics to study the effect of chitosan and carboxymethyl cellulose coatings on strawberries packaged in polyethylene terephthalate containers and stored at low temperatures. Their findings included the identification of a set of proteins related to browning, primarily associated with primary and secondary metabolism (Wang et al., 2017).

Beyond the linear sequence and three-dimensional structure of proteins, post-translational modifications (PTMs) greatly influence protein function and activity. For example, quantitative phosphoproteome analysis has shown that SN2 can reduce browning in fresh-cut potatoes by altering the phosphorylation levels of kinases. A network involving serine/arginine-rich proteins and mitogen-activated protein kinases has been proposed as a potential kinase-substrate interaction system that influences browning (Li et al., 2023).

### 3.3 Metabolome and postharvest browning studies

Metabolomics is the holistic study of metabolites within a biological system under defined conditions, providing a snapshot of the physiological state of an organism. These metabolites reflect the final products of gene expression and regulatory interactions, often showing a more direct correlation with observed phenotypes than mRNA or protein levels. As a branch of omics science, metabolomics is particularly effective for its comprehensive coverage of biological processes and its ability to link genotype to phenotype. Current metabolomics research employs targeted, widely targeted, and untargeted strategies, requiring the use of sensitive and precise techniques like mass spectrometry (MS) coupled with gas chromatography (GC), liquid chromatography (LC), or nuclear magnetic resonance (NMR) spectroscopy (Meng et al., 2022).

The role of metabolomics in understanding the browning of postharvest crops has gained significant momentum. It is instrumental in identifying and characterizing bioactive compounds and chemical markers that may influence the quality and shelf-life of produce (Qiu et al., 2021; Gao et al., 2022). For instance, a study on pomegranate storage identified key secondary metabolites involved in aril browning, particularly those related to the biosynthesis pathways of flavonoids, flavonols, and isoflavonoids, with a focus on phenylpropanoid biosynthesis (Shi et al., 2022). Metabolomics has also revealed that the metabolic profile of morel mushrooms changes substantially during storage, with increases in amino acids and fatty acids and decreases in soluble sugars, organic acids, and certain phenolic compounds (Gao et al., 2022). Comparative metabolomics between lettuce cultivars with varying browning rates has pinpointed metabolites implicated in the browning process, such as differences in lysophospholipid levels, phospholipase and lipoxygenase activity, and the presence of caffeoylquinic acid derivatives (García et al., 2017).

### 3.4 Integrated omics approaches applied in understanding postharvest browning

The integration of various omics datasets is pivotal for unraveling the complex molecular mechanisms of enzymatic browning in postharvest crops. By combining multi-omics data, such as genomics, transcriptomics, proteomics, and metabolomics, researchers can gain a comprehensive understanding of the molecular events that affect postharvest crop quality (Aiese Cigliano et al., 2022; Xie et al., 2023). Data integration is a sophisticated process that requires advanced computational methods to merge and analyze diverse omics datasets effectively. Current research often utilizes functional and statistical networks to facilitate the visualization and interpretation of these complex data relationships, aiming to identify key metabolic hubs associated with the browning process.

Recent studies have demonstrated the power of multi-omics approaches in pinpointing the primary factors contributing to browning during postharvest treatments and storage (Qiao et al., 2022; Romero et al., 2022). For example, a combined transcriptomic and metabolomic investigation into the browning of fresh-cut eggplant identified fluctuations in membrane phospholipid and unsaturated fatty acid metabolites, as well as changes in the expression of genes related to membrane lipid metabolism (Liu et al., 2022a). Similarly, the analysis of harvested litchi using both transcriptomic and proteomic techniques has provided insights into the regulation of the anthocyanin biosynthesis pathway during the browning process (Qu et al., 2021).

## 4 Prospects

This review has highlighted the potential applications of an integrated omics approach to study the browning reactions that occur during the storage and processing of postharvest crops. It offers an in-depth look at how omics integration can enhance the development of anti-browning treatments in postharvest management. Omics platforms, including those for transcriptomics, proteomics, and metabolomics, have proven to be more effective than traditional methods, providing a powerful suite of tools for elucidating molecular markers, regulatory networks, and genes involved in the browning reaction.

Despite the advantages, the application of diverse omics techniques comes with the substantial challenge of managing and making sense of the massive amounts of data they generate. Integrating this high-throughput data from various sources remains a daunting task, necessitating sophisticated analytical strategies. The successful integration of omics data will depend on the development of user-friendly analytical tools that are tightly linked to biological processes. Advanced data integration tools, such as machine learning should be considered to take advantage of omics datasets for constructing more biologically realistic network models across different biological layers from gene expression to postharvest browning reaction.

Metabolomics, in particular, faces hurdles such as the sheer complexity of the metabolome, gaps in our understanding of metabolic pathways, and difficulties in identifying molecules by their structural detector signals, compounded by the lack of extensive, metabolite-specific libraries. Moreover, due to significant advancements in redox proteomics, the iodoacetyl tandem mass tag (iodoTMT)-based redox proteomic approach has been successfully utilized for detecting redox-sensitive proteins during tomato fruit ripening (Wang et al., 2021). This approach can now be applied to gain a deeper understanding of the mechanism by which ROS-mediated oxidative post-translational modifications occur during the browning reaction in postharvest crops. There is a pressing need for further research in multi-omics to directly link biochemical activities to biomarkers and to advance our understanding of the postharvest browning process. Future studies should aim to fill these knowledge gaps and leverage the power of integrated omics for practical applications in crop postharvest biology.

## Author contributions

CW: Writing – original draft. LM: Writing – original draft. GZ: Data curation, Investigation, Writing – original draft. XY: Data curation, Investigation, Writing – original draft. BP: Data curation, Investigation, Writing – original draft. JC: Data curation, Investigation, Methodology, Writing – review & editing. BH: Conceptualization, Writing – original draft, Writing – review & editing. FS: Conceptualization, Writing – review & editing.

## References

[B1] Aiese Cigliano R. Aversano R. Di Matteo A. Palombieri S. Termolino P. Angelini C.. (2022). Multi-omics data integration provides insights into the post-harvest biology of a long shelf-life tomato landrace. Horticulture Res. 9, uhab042. doi: 10.1093/hr/uhab042 PMC8801724 35039852

[B2] Ali S. Sattar Khan A. Ullah Malik A. Anjum M. A. Nawaz A. Shoaib Shah H. M. (2019). Modified atmosphere packaging delays enzymatic browning and maintains quality of harvested litchi fruit during low temperature storage. Scientia Hortic. 254, 14–20. doi: 10.1016/j.scienta.2019.04.065

[B3] Ban Z. Yan J. Wang J. Zhang J. Yuan Q. Li L. (2018). Effects of postharvest application of chitosan-based layer-by-layer assemblies on regulation of ribosomal and defense proteins in strawberry fruit (fragaria x ananassa). Scientia horticulturae, 240. doi: 10.1016/j.scienta.2018.06.035

[B4] Coetzer C. Corsini D. Love S. Pavek J. Tumer N. (2001). Control of enzymatic browning in potato (Solanum tuberosum L.) by sense and antisense RNA from tomato polyphenol oxidase. J. Agric. Food Chem. 49 (2), 652–657. doi: 10.1021/jf001217f 11262007

[B5] Fan J. Du W. Chen Q.-L. Zhang J.-G. Yang X.-P. Hussain S. B.. (2021). Comparative transcriptomic analyses provide insights into the enzymatic browning mechanism of fresh-cut sand pear fruit. Horticulturae 7, (11). doi: 10.3390/horticulturae7110502

[B6] Feng X. An Y. Zheng J. Sun M. Wang L. (2016). Proteomics and SSH analyses of ALA-promoted fruit coloration and evidence for the involvement of a MADS-box gene, mdMADS1. Front. Plant Sci. 7. doi: 10.3389/fpls.2016.01615 PMC5098116 27872628

[B7] Gao F. Xie W. Zhang H. Li Z. Li S. Li T. (2022). Metabolomic analysis of browning mechanisms of morels (Morchella sextelata) during storage. Postharvest Biol. Technol. 185, 111801. doi: 10.1016/j.postharvbio.2021.111801

[B8] García C. J. García-Villalba R. Gil M. I. Tomas-Barberan F. A. (2017). LC-MS untargeted metabolomics to explain the signal metabolites inducing browning in fresh-cut lettuce. J. Agric. Food Chem. 65 (22), 4526–4535. doi: 10.1021/acs.jafc.7b01667 28506062

[B9] Hamdan N. Lee C. H. Wong S. L. Fauzi C. E. Zamri N. M. Lee T. H. (2022). Prevention of enzymatic browning by natural extracts and genome-editing: A review on recent progress. Molecules 27, (3). doi: 10.3390/molecules27031101 PMC8839884 35164369

[B10] He B. Hu F. Q. Du H. Y. Cheng J. J. Pang B. W. Chen X.. (2022). Omicsdriven crop potassium use efficiency breeding. Front. Plant Sci. 13. doi: 10.3389/fpls.2022.1076193 PMC9730507 36507409

[B11] Li M. Hong Y. Wang X. (2009). Phospholipase D- and phosphatidic acid-mediated signaling in plants. Biochim. Biophys. Acta (BBA) - Mol. Cell Biol. Lipids 1791 (9), 927–935. doi: 10.1016/j.bbalip.2009.02.017 19289179

[B12] Li M. Li X. Han C. Ji N. Jin P. Zheng Y. (2019b). Physiological and metabolomic analysis of cold plasma treated fresh-cut strawberries. J. Agric. Food Chem. 67 (14), 4043–4053. doi: 10.1021/acs.jafc.9b00656 30883111

[B13] Li M. Li X. Han C. Ji N. Jin P. Zheng Y. (2019c). UV-C treatment maintains quality and enhances antioxidant capacity of fresh-cut strawberries. Postharvest Biol. Technol. 156, 110945. doi: 10.1016/j.postharvbio.2019.110945

[B14] Li L.-Q. Mu Y.-L. Chen J. Wang Q. Lu Y.-F. Xin S.. (2023). Molecular mechanism by which StSN2 overexpression inhibits the enzymatic browning of potato. Postharvest Biol. Technol. 203, 112416. doi: 10.1016/j.postharvbio.2023.112416

[B15] Li J. Zhou X. Wei B. Cheng S. Zhou Q. Ji S. (2019a). GABA application improves the mitochondrial antioxidant system and reduces peel browning in ‘Nanguo’ pears after removal from cold storage. Food Chem. 297, 124903. doi: 10.1016/j.foodchem.2019.05.177 31253345

[B16] Lin Y. Chen G. Lin H. Lin M. Wang H. Lin Y. (2020). Chitosan postharvest treatment suppresses the pulp breakdown development of longan fruit through regulating ROS metabolism. Int. J. Biol. Macromolecules 165, 601–608. doi: 10.1016/j.ijbiomac.2020.09.194 33002534

[B17] Lin Y. Lin H. Lin Y. Zhang S. Chen Y. Jiang X. (2016). The roles of metabolism of membrane lipids and phenolics in hydrogen peroxide-induced pericarp browning of harvested longan fruit. Postharvest Biol. Technol. 111, 53–61. doi: 10.1016/j.postharvbio.2015.07.030

[B18] Liu B. Fang F. Guan H. Zhang J. Luo H. Zhong R.. (2024). Integrated function of proanthocyanidin and lignin polymerization mediated by LAC/PRXs in pericarp browning of longan fruit. Postharvest Biol. Technol. 207, 112618. doi: 10.1016/j.postharvbio.2023.112618

[B19] Liu F. Huang W. Feng Z. Tao Y. Fan Y. He W.. (2021a). Proteomic analyses on the browning of shade-dried Thompson seedless grape. Appl. Biol. Chem. 64 (1), 41. doi: 10.1186/s13765-021-00612-7

[B20] Liu Y. Liao L. Yin F. Song M. Shang F. Shuai L.. (2022b). Integration of metabolome and transcriptome profiling reveals the effect of 6-Benzylaminopurine on the browning of fresh-cut lettuce during storage. Postharvest Biol. Technol. 192, 112015. doi: 10.1016/j.postharvbio.2022.112015

[B21] Liu X. Xiao K. Zhang A. Zhu W. Zhang H. Tan F.. (2022a). Metabolomic analysis, combined with enzymatic and transcriptome assays, to reveal the browning resistance mechanism of fresh-cut eggplant. Foods 11, (8). doi: 10.3390/foods11081174 PMC9031582 35454761

[B22] Liu X. Zhang A. Shang J. Zhu Z. Li Y. Wu X.. (2021b). Study on browning mechanism of fresh-cut eggplant (Solanum melongena L.) based on metabolomics, enzymatic assays and gene expression. Sci. Rep. 11 (1), 6937. doi: 10.1038/s41598-021-86311-1 33767263 PMC7994816

[B23] Liu X. Zhang A. Zhao J. Shang J. Zhu Z. Wu X.. (2021c). Transcriptome profiling reveals potential genes involved in browning of fresh-cut eggplant (Solanum melongena L.). Sci. Rep. 11 (1), 16081. doi: 10.1038/s41598-021-94831-z 34373468 PMC8352891

[B24] Loizzo M. R. Tundis R. Menichini F. (2012). Natural and synthetic tyrosinase inhibitors as antibrowning agents: an update. Compr. Rev. Food Sci. Food Saf. 11 (4), 378–398. doi: 10.1111/j.1541-4337.2012.00191.x

[B25] Lou C. P. He B. Shi P. B. Xi J. L. Gui H. B. Pang B. W.. (2022). Transcriptome dynamics uncovers long non-coding RNAs response to salinity stress in *Chenopodium quinoa* . Front. Plant Sci. 13. doi: 10.3389/fpls.2022.988845 PMC9530330 36204077

[B26] Ma W. Li J. Murtaza A. Iqbal A. Zhang J. Zhu L.. (2022). High-pressure carbon dioxide treatment alleviates browning development by regulating membrane lipid metabolism in fresh-cut lettuce. Food Control 134, 108749. doi: 10.1016/j.foodcont.2021.108749

[B27] Mathabe P. M. K. Belay Z. A. Ndlovu T. Caleb O. J. (2020). Progress in proteomic profiling of horticultural commodities during postharvest handling and storage: A review. Scientia Hortic. 261, 108996. doi: 10.1016/j.scienta.2019.108996

[B28] Meng L. Song W. J. Chen S. W. Hu F. Q. Pang B. W. Cheng J. J.. (2022). Widely targeted metabolomics analysis reveals the mechanism of quality improvement of flue-cured tobacco. Front. Plant Sci. 13. doi: 10.3389/fpls.2022.1074029 PMC9746875 36523627

[B29] Min T. Lu K. Chen J. Niu L. Lin Q. Yi Y.. (2023). Biochemical mechanism of fresh-cut lotus (Nelumbo nucifera gaertn.) root with exogenous melatonin treatment by multiomics analysis. Foods 12, (1). doi: 10.3390/foods12010044 PMC9818798 36613262

[B30] Moon K. M. Kwon E.-B. Lee B. Kim C. Y. (2020). Recent trends in controlling the enzymatic browning of fruit and vegetable products. Molecules 25, (12). doi: 10.3390/molecules25122754 PMC7355983 32549214

[B31] Nokthai P. Lee V. S. Shank L. (2010). Molecular modeling of peroxidase and polyphenol oxidase: substrate specificity and active site comparison. Int. J. Mol. Sci. 11 (9), 3266–3276. doi: 10.3390/ijms11093266 20957092 PMC2956093

[B32] Pandit A. A. Shah R. A. Husaini A. M. Transcriptomics: A time-efficient tool with wide applications in crop and animal biotechnology. J. Pharm. Phyt. 2018;7 (2):1701–1704.

[B33] Parveen I. Threadgill M. D. Moorby J. M. Winters A. (2010). Oxidative phenols in forage crops containing polyphenol oxidase enzymes. J. Agric. Food Chem. 58 (3), 1371–1382. doi: 10.1021/jf9024294 20078064

[B34] Paudel P. Seong S. H. Wagle A. Min B. S. Jung H. A. Choi J. S. (2020). Antioxidant and anti-browning property of 2-arylbenzofuran derivatives from Morus alba Linn root bark. Food Chem. 309, 125739. doi: 10.1016/j.foodchem.2019.125739 31787394

[B35] Qiao L. Gao M. Wang Y. Tian X. Lu L. Liu X. (2022). Integrated transcriptomic and metabolomic analysis of cultivar differences provides insights into the browning mechanism of fresh-cut potato tubers. Postharvest Biol. Technol. 188, 111905. doi: 10.1016/j.postharvbio.2022.111905

[B36] Qiu S. Tu Y. Huang D. Dong Z. Huang M. Cheng J.. (2021). Selection of appropriate post-harvest processing methods based on the metabolomics analysis of Salvia miltiorrhiza Bunge. Food Res. Int. 144, 110366. doi: 10.1016/j.foodres.2021.110366 34053559

[B37] Qu S. Li M. Wang G. Yu W. Zhu S. (2021). Transcriptomic, proteomic and LC-MS analyses reveal anthocyanin biosynthesis during litchi pericarp browning. Scientia Hortic. 289, 110443. doi: 10.1016/j.scienta.2021.110443

[B38] Qu H. Zhou H. Ma T. Zheng Z. Zheng E. Yang H.. (2022). TMT-based quantitative proteomic analysis of postharvest Coprinus comatus fruiting body during storage. Postharvest Biol. Technol. 185, 111786. doi: 10.1016/j.postharvbio.2021.111786

[B39] Rezayian M. Niknam V. Ebrahimzadeh H. (2019). Oxidative damage and antioxidative system in algae. Toxicol. Rep. 6, 1309–1313. doi: 10.1016/j.toxrep.2019.10.001 31993331 PMC6978204

[B40] Romero I. Escribano M. I. Merodio C. Sanchez-Ballesta M. T. (2022). Postharvest high-CO2 treatments on the quality of soft fruit berries: an integrated transcriptomic, proteomic, and metabolomic approach. J. Agric. Food Chem. 70 (28), 8593–8597. doi: 10.1021/acs.jafc.2c01305 35792090 PMC9305969

[B41] Schieber A. (2018). Reactions of quinones—Mechanisms, structures, and prospects for food research. J. Agric. Food Chem. 66 (50), 13051–13055. doi: 10.1021/acs.jafc.8b05215 30472845

[B42] Shi J. Wang S. Tong R. Wang S. Chen Y. Wu W.. (2022). Widely targeted secondary metabolomics explored pomegranate aril browning during cold storage. Postharvest Biol. Technol. 186, 111839. doi: 10.1016/j.postharvbio.2022.111839

[B43] Shrestha L. Kulig B. Moscetti R. Massantini R. Pawelzik E. Hensel O.. (2020). Optimisation of physical and chemical treatments to control browning development and enzymatic activity on fresh-cut apple slices. Foods 9, (1). doi: 10.3390/foods9010076 PMC7022590 31936660

[B44] Singh B. Singh J. P. Kaur A. Singh N. (2016). Bioactive compounds in banana and their associated health benefits - A review. Food Chem. 206, 1–11. doi: 10.1016/j.foodchem.2016.03.033 27041291

[B45] Singh B. Singh J. P. Kaur A. Singh N. (2017). Phenolic composition and antioxidant potential of grain legume seeds: A review. Food Res. Int. 101, 1–16. doi: 10.1016/j.foodres.2017.09.026 28941672

[B46] Sirangelo T. M. Rogers H. J. Spadafora N. D. (2022). Multi-omic approaches to investigate molecular mechanisms in peach post-harvest ripening. Agriculture 12, (4). doi: 10.3390/agriculture12040553

[B47] Sui X. Meng Z. Dong T. Fan X. Wang Q. (2023). Enzymatic browning and polyphenol oxidase control strategies. Curr. Opin. Biotechnol. 81, 102921. doi: 10.1016/j.copbio.2023.102921 36965297

[B48] Sun Y. Luo M. Ge W. Zhou X. Zhou Q. Wei B.. (2022). Phenylpropanoid metabolism in relation to peel browning development of cold-stored ‘Nanguo’ pears. Plant Sci. 322, 111363. doi: 10.1016/j.plantsci.2022.111363 35750293

[B49] Sun Y. Sun H. Luo M. Zhou X. Zhou Q. Wei B.. (2020). Membrane lipid metabolism in relation to core browning during ambient storage of ‘Nanguo’ pears. Postharvest Biol. Technol. 169, 111288. doi: 10.1016/j.postharvbio.2020.111288

[B50] Tinello F. Lante A. (2018). Recent advances in controlling polyphenol oxidase activity of fruit and vegetable products. Innovative Food Sci. Emerging Technol. 50, 73–83. doi: 10.1016/j.ifset.2018.10.008

[B51] Trabelsi H. Cherif O. A. Sakouhi F. Villeneuve P. Renaud J. Barouh N.. (2012). Total lipid content, fatty acids and 4-desmethylsterols accumulation in developing fruit of Pistacia lentiscus L. growing Wild Tunisia. Food Chem. 131 (2), 434–440. doi: 10.1016/j.foodchem.2011.08.083

[B52] Wang P. Li X. Wang Y. Wang W. Tian S. Qin G. (2021). Redox proteomic analysis reveals the involvement of oxidative post-translational modification in tomato fruit ripening. Postharvest Biol. Technol. 178, 111556. doi: 10.1016/j.postharvbio.2021.111556

[B53] Wang L. Tang T. Wang W. Zhang J. Wang Z. Wang F. (2022). Multi-omics landscape of DNA methylation regulates browning in “Fuji” Apple. Front. Nutr. 8. doi: 10.3389/fnut.2021.800489 PMC8859415 35198585

[B54] Wang L. Wang W. Shan J. Li C. Suo H. Liu J.. (2023a). A genome-wide view of the transcriptome dynamics of fresh-cut potato tubers. Genes 14, (1). doi: 10.3390/genes14010181 PMC9859442 36672922

[B55] Wang X. Zhang X. Jia P. Luan H. Qi G. Li H.. (2023b). Transcriptomics and metabolomics provide insight into the anti-browning mechanism of selenium in freshly cut apples. Front. Plant Sci. 14. doi: 10.3389/fpls.2023.1176936 PMC10200898 37223812

[B56] Wang J.-W. Zhou X. Zhou Q. Liu Z.-Y. Sheng L. Wang L.. (2017). Proteomic analysis of peel browning of ‘Nanguo’ pears after low-temperature storage. J. Sci. Food Agric. 97 (8), 2460–2467. doi: 10.1002/jsfa.8060 27696427

[B57] Waszczak C. Akter S. Jacques S. Huang J. Messens J. Van Breusegem F. (2015). Oxidative post-translational modifications of cysteine residues in plant signal transduction. J. Exp. Bot. 66 (10), 2923–2934. doi: 10.1093/jxb/erv084 25750423

[B58] Winters A. L. Minchin F. R. Michaelson-Yeates T. P. T. Lee M. R. F. Morris P. (2008). Latent and active polyphenol oxidase (PPO) in red clover (Trifolium pratense) and use of a low PPO mutant to study the role of PPO in proteolysis reduction. J. Agric. Food Chem. 56 (8), 2817–2824. doi: 10.1021/jf0726177 18361497

[B59] Xie Y. Lv Y. Jia L. Zheng L. Li Y. Zhu M.. (2023). Plastid-localized amino acid metabolism coordinates rice ammonium tolerance and nitrogen use efficiency. Nat. Plants. 9 (9), 1514–1529. doi: 10.1038/s41477-023-01494-x 37604972

[B60] Yi C. Jiang Y. Shi J. Qu H. Duan X. Yang B.. (2009). Effect of adenosine triphosphate on changes of fatty acids in harvested litchi fruit infected by Peronophythora litchii. Postharvest Biol. Technol. 54 (3), 159–164. doi: 10.1016/j.postharvbio.2009.06.008

[B61] Yun Z. Li T. Gao H. Zhu H. Gupta V. K. Jiang Y.. (2019). Integrated transcriptomic, proteomic, and metabolomics analysis reveals peel ripening of harvested banana under natural condition. Biomolecules 9, (5). doi: 10.3390/biom9050167 PMC6572190 31052343

[B62] Zhao P. Li W. Zhen C. Wang K. Qin Z. Gao H. (2021). Transcriptomic analysis of the effects of γ-aminobutyric acid treatment on browning and induced disease resistance in fresh-cut apples. Postharvest Biol. Technol. 181, 111686. doi: 10.1016/j.postharvbio.2021.111686

[B63] Zhou Y. Chen Z. He M. Gao H. Zhu H. Yun Z.. (2020b). Unveiling the complexity of the litchi transcriptome and pericarp browning by single-molecule long-read sequencing. Postharvest Biol. Technol. 168, 111252. doi: 10.1016/j.postharvbio.2020.111252

[B64] Zhou H. Tian M. Huang W. Luo S. Hu H. Zhang Y.. (2020a). Physiological and transcriptomic analysis of ‘Whangkeumbae’ pear core browning during low-temperature storage. Gene Expression Patterns 36, 119113. doi: 10.1016/j.gep.2020.119113 32325218

[B65] Zhu L. Hu W. Murtaza A. Iqbal A. Kong M. Zhang J.. (2023). Browning inhibition in fresh-cut Chinese water chestnut under high pressure CO2 treatment: Regulation of reactive oxygen species and membrane lipid metabolism. Food Chem. 427, 136586. doi: 10.1016/j.foodchem.2023.136586 37399645

